# Perspectives of Healthcare Providers towards Remote Medical Interpreting Services in Japan

**DOI:** 10.3390/healthcare11010112

**Published:** 2022-12-30

**Authors:** Soichiro Saeki, Kaori Minamitani, Fumika Iwaoka, Kokoro Shirai

**Affiliations:** 1 Center Hospital of the National Center for Global Health and Medicine, Tokyo 162-8655, Japan; 2 Department of Global and Innovative Medicine, Graduate School of Medicine, Osaka University, Osaka 565-0871, Japan; 3 Department of International Medical Care, Rinku General Medical Center, Osaka 559-8611, Japan; 4 Department of Public Health, Graduate School of Medicine, Osaka University, Osaka 565-0871, Japan; 5 Institute for Global Health Policy Research, Bureau of International Health Cooperation, National Center for Global Health and Medicine, Tokyo 162-8655, Japan

**Keywords:** distance interpreting, medical interpreters, healthcare interpreters, telecommunication, migrant health, minority health, global health, non-national patients, foreigners, emigrants and immigrants

## Abstract

Language support is necessary for effective healthcare as language obstacles have a negative impact on patient outcomes. Medical facilities dealing with novel coronavirus disease (COVID-19) were forced to restrict the number of healthcare professionals on the field, and medical interpreters were no exception. This has prompted the introduction of remote medical interpreting systems, which do not necessitate the presence of an interpreter onsite. However, as the dominant trend in offering linguistic help was face-to-face medical interpreting, healthcare staff are also battling with its utilization. We conducted a single-centered, retrospective study by examining written responses taken from April 2018 to March 2020 and a total of 236 healthcare employees in Japan, to identify the primary reasons of such challenges. Remote medical interpreting was frequently employed by a range of professions in many departments across various languages, and the majority of users were satisfied with the experience. The qualitative analysis based on the free opinions of the healthcare professionals unraveled three main concerns towards remote medical interpreting: connection to the interpreting providers; coordination of the remote interpreting coordinators, and quality of interpreting. Therefore, increasing the use of remote medical interpreting while simultaneously training interpreters by enhancing the skills required in Japanese medical facilities would be necessary.

## 1. Introduction

Migrant health has now become a worldwide issue in global health, even in high-income countries such as Japan [1]. However, the health care of patients without a Japanese nationality has been underrecognized until recently, when the Japanese government began to introduce measures to deal with the increasing number of foreign tourists. This includes providing linguistic assistance to patients with limited Japanese proficiency (LJP) [2] as well as accrediting certain medical facilities that are capable of providing healthcare to foreigners.

However, linguistic barriers still pose as a problem in medical care for LJPs in Japan [3]. According to a survey conducted to foreign residents in Japan, more than 60% answered that they were in fear of communication problems due to the lack of linguistic ability [4]. The situation is far more severe among medical staff; up to 95% of the hospitals in Japan answered that they were worried of linguistic issues if they were to deal with foreign patients, according to a national survey conducted by the Japan Hospital Association [5]. Previous studies conducted on foreign students in Japan identified that linguistic barriers indeed hinder them from receiving medical care [6], while number of Japanese peers correlated with the increase in health promotion behaviors [7], which also raises awareness for students unable to communicate properly on their own. In other words, healthcare professionals and patients were both deeply concerned of communication errors due to linguistic issues.

Linguistic assistance is essential when providing adequate healthcare. In order to provide LJP patients the same quality of healthcare in Japan [8], the Ministry of Health, Labour and Welfare began to accredit certified medical interpreters, in assistance of the International Society for Clinical Medicine in 2019 [2]. This trend has assisted fostering training of such human resources and the recognition of such tasks, but the implementation of medical interpreters is far from common in Japanese hospitals. Although hospitals that have the capacity of taking care of foreign patients are certified in Japan Medical Service Accreditation for International Patients (JMIP, Japan Medical Education Foundation, Tokyo, Japan), organized by the Ministry of Health, Labour and Welfare from 2012 [9], the number of hospitals that provide daily medical interpreting services are still a minority [5]. Possible reasons behind this are the lack of certified medical interpreters in Japan, and the initial exceeding costs needed to hire the interpreters [10,11].

This problem may be solved with the utilization of remote (mobile) medical interpreting, by connecting with remote medical interpreters over telephone or video calls [12,13]. This enables hospitals to deal with patients with linguistic barriers, even for hospitals that hire medical interpreters on site, for the medical facilities that lack such resources [10]. Utilization of such resources is the key, initial step in providing necessary preparations to deal with the rising need for medical services for non-Japanese patients.

The study of remote medical interpreting in Japan, however, has not received much attention to date. On the other hand, such studies have been widely conducted throughout the world. The preference [14,15,16] and quality [17,18,19,20,21,22] of on-site and remote medical interpreters varies between previous studies, as well as between video and telephone interpreting [23,24,25,26]. In addition, during the novel coronavirus disease (COVID-19) pandemic, remote medical interpreting has emerged as a viable option for linguistic assistance [27]. Hospitals restricted people’s access during COVID-19 pandemic, including patients and their families [28], and medical staff, such as interpreters [27] and medical trainees [29,30]. Even while travel limitations made it difficult for foreign tourists to visit other nations, foreign residents [31] who struggle with language challenges nonetheless require medical care, and studies revealed that remote medical interpreting would serve as a feasible linguistic assistance tool [27].

Given that the Japanese language is high-context [32] compared to other Western languages, the benefits or barriers in the use of linguistic services such as medical interpreters may differ from other nations. Therefore, we studied the perspectives of medical professionals towards remote medical interpreting in the setting of a Japanese medical facility to identify the key requirements to enhance the implementation of remote medical interpreting in Japan.

## 2. Materials and Methods

This study is a qualitive and quantitative study conducted in a single Japanese hospital, mainly prior to the COVID-19 pandemic.

### 2.1. Study Design and Setting

This study is a single-center, retrospective observational study that took place in Rinku General Medical Center (RGMC, Izumisano, Osaka, Japan). RGMC is a general medical center with a variety of medical care from general internal medicine to tertiary care, located across from Kansai International Airport (KIX), one of the largest international hub airports in Japan. This nature of the location has led to many foreign patients to visit RGMC [31,33]. RGMC has a department that specializes in care for foreign patients, with medical interpreters available in English, Chinese, Portuguese and Spanish, and assistance in other languages are available with the use of remote medical interpreting, such as video calls and telephones. These facilities have attracted many foreign residents to attend RGMC, which is certified by JMIP from 2013 [34].

### 2.2. Subjects

Of all the patients that visited RGMC during April 2018 to March 2020, medical staff who used remote medical interpreting services (telephone or video calls) were included. Medical staff in RGMC were required to fill in a feedback sheet (described in detail in Section 2.3) when using remote medical interpreting services, and therefore all staff members using remote medical interpreting were recruited for the study. As the primary medical interpreting service provider for RGMC changed after April 2020 (the fiscal year of Japan), data of medical interpreting services after April 2020 were excluded from this study.

As this study is a retrospective, observational study using previous medical records, opt-out consent was determined using the official website of RGMC. This study protocol was approved by the Ethics committee of Rinku General Medical Center (approval number 21-004).

### 2.3. Data Curation

Medical staff using remote medical interpreting were required to report on its usage in a written format with six questions described below (originally in Japanese) per every use, and feedback about its use was obtained simultaneously. From the report, descriptive data of the user, language, department and situation the interpreting service was used, and feedback regards to the quality of the service was obtained. This was primarily obtained in order to provide feedback to Mediway (Towa Engineering, Tokyo, Japan), the service used for remote medical interpreting in RGMC.

The reports included the date and time of use, interpreting language, location of use, evaluator attributes (e.g., job title), content of interpretation, in-hospital identification number (ID) of the patient who used the service, and interpretation method (video interpretation, telephone interpretation (cell phone or PHS (hospital pager)). In addition, as feedback, they were asked six questions: (1) Did you encounter any problems with the use of the service? If so, please indicate what problems you had; (2) How would you rate the access time of the interpreter: short, normal, long; (3) How would you rate the audio quality: good, normal, hard to hear; (4) How would you rate the video quality (for video interpretation): good visual, normal, bad visual; (5) Did you encounter any of the following?: often had to ask patients and staff back, polite and neat responses, problems with response, fluent and smooth interpreting; and (6) Any additional comments you would like to make? The answers were collected in Japanese, and was translated by the authors into English.

Data of the patients such as nationality, language used for translation was obtained from the records kept by the International Outpatient Department of RGMC. This record is kept independently from other medical records, and is handled by the international medical coordinators of RGMC.

### 2.4. Statistical Analysis

We presented each categorical variable is represented with numbers and percentages. *p* values were computed using Fisher’s exact test for items with five or more in either group and the Chi-square test for items with five or fewer. Statistical analysis was performed using EZR (Saitama Medical Center, Jichi Medical University, Saitama, Japan), a graphical user interface for R [35]. The R version used was 3.6.1 (R core team, Vienna, Austria) [36]. The *p*-value < 0.05 was determined as statistically significant.

The open-ended responses in the report were transcribed as data and coded using content analysis [37]. The coded content was checked by the co-authors and classified into categories. Such procedures were conducted in Japanese, and the results were translated into English.

## 3. Results

We hereby present the results of the qualitive data of the demographic features of the implementation of remote medical interpreting in RGMC, and quantitative data of the perspectives of healthcare workers towards medical interpreting.

### 3.1. Usage of Remote Medical Interpreting

During April 2018 to March 2020, a total of 236 health care providers used remote medical interpreting in RGMC. The majority of the use was telephone interpreting with using the specified cell phone followed by video interpreting, which covered 78.2% and 20.1%, respectively (Table 1).

The trend of the languages used by the medical interpreting services during this period is described in Table 2. The largest use was among the Chinese language (42.7%), while other languages used in nearby Association of South East Asian Nations (ASEAN) countries mainly covered the rest of the languages.

The location that used remote medical interpreting the most was the emergency department, which totaled over 40% when combining the emergency room (35.1%) and the tertiary care center (5.6%). The obstetrics and gynecology (OBGYN) department and general internal medicine followed with 26.7% and 5.2%, respectively (Table 3). Of the 236 usages, interpretation was used in consultations 138 times (68.0%), 95 times (46.8%) when explaining the results or gaining consent, and 21 times (10.3%) for making payments.

### 3.2. Feedback

Regarding to the feedbacks, the evaluators were mainly nurses (38.3%), followed by the international medical coordinators, the secretarial staff appointed specifically to coordinate medical care for international patients (30.6%). Feedback from physicians were limited to 23.3% (Table 4).

The evaluators rated the quality of the audio and interpreter fairly (Table 5). Over 90% of the evaluators reported the quality of audio to be equal to or better than normal. 57.2% of the evaluators marked “polite and neat responses”, while there were only three (1.4%) reports of “problems with response”.

The comments from the healthcare professionals who evaluated the interpreters were classified into three categories: (1) connection with the remote medical interpreters/interpreting system; (2) the coordination of the remote medical interpreting company; and (3) the quality of the interpreters (Table 6). Inadequate connection to the medical interpreting system hindered proper use of medical interpreting, especially when the evaluators commented the quality of the audio to be poor. Several evaluators reported not being able to use the medical interpreting service, as the company was unable to assign the interpreter of the requested language. The largest comments in detail were regarding to the quality of the interpreters. The evaluators were especially concerned about the usage of difficult medical terms, expressing anxiety when unable to be certain if the patient could completely understand the contents of the conversations, regardless of the department or situation the interpreting service was used. The comments presented in Table 6 were either from nurses or the international coordinators.

## 4. Discussion

Our study reported the demographic usage of remote medical interpreting in a Japanese hospital, and revealed several concerns of healthcare providers encountered during its use. To the best of our knowledge, no prior study has been reported in Japan to present data on perspectives on remote medical interpreting, thus focused on medical interpreting in the Japanese language.

The main objective of our study was to identify the unique perspectives of healthcare providers in Japan towards remote medical interpreting. Most users recruited in our study seemed to be satisfied with the usage of remote medical interpreting services, which has been recognized as an uprising trend during the COVID-19 pandemic [27]. Furthermore, our study identified that not only the quality of the interpreters but also the coordination quality of the remote medical interpreting agency was also linked with satisfaction, which has not been widely reported in previous studies throughout the world.

The usability of remote medical interpreting reported in our study seemed to be smooth, which complies with previous studies [23]. Another study did not report a significant difference in technical issues between video and telephone interpreting [38], but the lack of telephone interpreting cases hindered us from making an adequate comparison in our study. As presented, many different languages were required to be translated from Japanese. As remote interpreting is reported to be useful in situations where various languages are necessary [12], healthcare professionals in RGMC may have found this service to be useful.

However, anxiety was presented by healthcare providers under several circumstances. Inadequate networking technology with bad connection to telephone connections will cut off conversations by creating pauses. This is inevitable in several areas of the hospital, as there are many thick walls that separate each room. Building new connection points in such specific areas should increase connectivity, but such investments should be considered with the cost–benefits, by confirming how often and how critically medical interpreting is needed in such locations.

In addition, inadequate coordination by the remote medical interpreting companies may lead to the loss of opportunity to intervene in patients with limited Japanese proficiency. There are several situations where companies were unable to provide medical interpreters. One is when the medical interpreters of the company have already been occupied in other interpreting missions. Another possibility is that the interpreters for a specific language are off duty. This may occur more in languages with few speakers. Although we recognize that emergency situations occur in medical facilities, we recommend medical professionals to book an appointment with medical interpreters prior to the consultations using languages with few speakers.

Furthermore, the lack of quality of the medical interpreters became the key stressors when remote medical interpreting was actually in use. In Japan, it is not mandatory for medical interpreters to acquire the qualification as medical interpreters, and it remains uncertain whether every medical interpreter has gone through proper training. Especially for languages with only few speakers, it would be difficult to maintain proper quality, as human resources become scarce. In addition, healthcare providers who use remote medical interpreting services should be guided to provide necessary information to the interpreters. For example, consultations and check-ups in the OBGYN will require intense communication with many instructions. This factor may be of greater importance in the high-context Japanese language, where the speaker often omits the subject of the sentence. If the interpreter cannot understand the situation, they may not be able to provide the necessary instructions. In other words, both medical interpreters and medical professionals require training to reach the best results in the use of remote medical interpreting.

Although this novel study conducted in Japan has many strengths with the availability of data that no other hospitals may have, several limitations should be acknowledged.

Firstly, medical records of interpreting were kept based upon reports of medical professionals that used the service. Therefore, the records may not be completely accurate. Secondly, as this is a retrospective study using written records, some cases where medical professionals used other services by themselves, such as smartphone applications on their own mobile phones may not have been included in this study. For the same reason, phone inquiries made by the patients have not been included, if the patient did not come to the hospital for care. However, these situations should be minimal, which should not be enough to compromise the results of this study. Finally, this study was conducted in a single medical center in Japan. Interpreters and healthcare professionals both improved their skills simultaneously, and thus similar studies among different medical facilities and medical interpreting companies should be conducted.

This study was not able to compare on-site medical interpreting and remote medical interpreting directly to analyze the exact preference of medical professionals in Japan. Additional research is also required to capture the voices of patients who use remote medical interpreting services, as well as the influence of the patients’ demographic features on the outcomes presented in our study. As foreign patients may face not only linguistic but also legislative and financial difficulties in the medical facilities [39], this may also influence the difficulty in communication, which may impact the medical staff’s necessity towards such linguistic assistance. Although gathering information from patients who do not speak the native language has been difficult [40], a new legislative amendment has been adopted in 2022 to encourage such studies that promote public health in Japan [41]. We are currently seeking new research strategies to be able to conduct such studies in the near future.

## 5. Conclusions

Overall, Japanese medical professionals rated remote medical interpreting positively. However, further improvement in its quality is necessary to provide a higher care standard for non-Japanese speaking patients in Japan.

## Figures and Tables

**Table 1:** Interpreting method used for remote medical interpreting.

		*n* = 236 (%)
Interpreting method	Specified cellphone	179 (78.2)
	Video Interpreting	46 (20.1)
	PHS (hospital pager)	4 (1.7)
	Unanswered	7 (2.9)

**Table 2:** Languages used for remote medical interpreting.

		*n* = 236 (%)
Languages	Chinese	100 (42.7)
	Vietnamese	38 (16.2)
	Indonesian	22 (9.4)
	English	16 (6.8)
	Portuguese	10 (4.3)
	Burmese	8 (3.4)
	Thai	7 (3.0)
	Korean	6 (2.6)
	French	6 (2.6)
	Nepalese	5 (2.1)
	Sinhala	5 (2.1)
	Others ^1^	11 (4.7)

^1^ “Others” included Persian, German, Tagalog and Russian.

**Table 3:** Locations and situations of the use of remote medical interpreting.

		*n* = 236 (%)
Locations	Emergency Department	81 (35.1)
	OBGYN	63 (26.7)
	Tertiary Emergency Center	13 (5.6)
	General Internal Medicine	12 (5.2)
	Pediatrics	7 (3.0)
	Urology	4 (1.7)
	EICU	3 (1.3)
	Plastic Surgery	3 (1.3)
	General reception	3 (1.3)
	Unspecified	35 (14.8)
Situations	Consultations	138 (68.0)
	Explaining results	95 (46.8)
	Payments	21 (10.3)

Table legends: OBGYN obstetrics and gynecology; EICU emergency intensive care unit.

**Table 4:** Demographic data of the evaluators.

		*n* = 236 (%)
Gender	Female	126 (53.4)
	Male	79 (33.5)
	Unanswered	31 (13.1)
Attributes	Nurse	85 (38.3)
	International Medical Coordinator	68 (30.6)
	Physician	53 (23.9)
	Midwife	12 (5.4)
	Speech Therapist	1 (0.5)
	Physical Therapist	1 (0.5)
	Administration staff	1 (0.5)
	Clerk	1 (0.5)
	Unanswered	19 (8.1)

**Table 5:** Evaluation of remote medical interpreting by healthcare providers.

		*n* = 236 (%)
Audio quality	Good	85 (38.3)
	Normal	117 (52.7)
	Hard to hear	17 (7.7)
	Others	2 (0.9)
	Unanswered	1 (0.5)
Video quality	Good visual	24 (10.8)
	Normal	31 (14.0)
	Bad visual	1 (0.5)
	Others	2 (0.9)
	Unanswered	164 (73.9)
Interpreter quality	Polite and neat responses	127 (57.2)
	Fluent and smooth interpreting	75 (33.8)
	Often had to ask patients and staff back	18 (8.1)
	Problems with response	3 (1.4)

**Table 6:** Feedback of the healthcare providers to the remote medical interpreting service.

Category	Codes	Comments
Connection	Technical errors	Audio and video are disconnected.
		They said it was hard to hear my voice over there. It is hard to talk loudly with a mask on.
		Poor network connection. The interpretation function could not be fulfilled, and the interpreting service itself was cancelled.
Coordination	Difficulty finding an interpreter	The second time, Chinese was not immediately available and kept the patient waiting for about 30 min.
		I waited for an interpreter but could not get through. The patient’s husband was Japanese, so I explained to him in Japanese and he offered to cancel the interpretation.
Quality of interpreter	Lack of smooth communication	The interpreter took too much time to speak, which interfered with smooth conversation.
		Bad rhythm. Didn’t understand the contents several times.
		I felt that training for interpreters is necessary. Did it take time for the patient to look up the meaning in a dictionary?
		Please provide more training. The patient and the medical staff felt uneasy because the calls such as, “The bed is going to move” were not smooth.
	Lack of medical knowledge	Several times the meaning of Japanese words such as gallstones, pain radiating to the backside, etc. were not understood and the doctor had to explain for the interpreter.
		I explained that a coronary artery aneurysm puts the patient at risk of having a heart attack, and he asked me if this was dangerous or not. I don’t know if the interpreter did not understand that risk or if the interpreter’s way of communicating was insufficient, although I interpreted exactly as he did, but it made me a little uneasy.
	Lack of communication with medical staff	Difficulty in communicating with the interpreter.
		The interpreter spoke in Chinese all the time and did not return Japanese until we asked him/her to translate into Japanese.

## Data Availability

Data for this study cannot be shared due to the confidentiality of the patients’ personal information.
